# Review of current best practices for human milk banking

**DOI:** 10.1111/mcn.13657

**Published:** 2024-05-16

**Authors:** Sharon L. Unger, Deborah L. O'Connor

**Affiliations:** ^1^ Department of Pediatrics Sinai Health Toronto Ontario Canada; ^2^ Department of Nutritional Sciences University of Toronto Toronto Ontario Canada; ^3^ Rogers Hixon Ontario Human Milk Bank Toronto Ontario Canada; ^4^ Division of Neonatology IWK Hospital Halifax Nova Scotia Canada; ^5^ Translational Medicine The Hospital for Sick Children Toronto Ontario Canada

**Keywords:** donor milk, human milk, milk bank, preterm infant

## Abstract

Mother's/parent milk is the optimal way to feed infants and when unavailable, supplemental donor human milk is preferred. A safe supply of donor human milk should be available for all low birthweight infants for whom it has been shown to reduce morbidity. Human milk banking has been in existence for more than a century, although largely shut down during the 1980s, primarily due to fears of human immunodeficiency virus transmission. With renewed security in milk banking, has come an exponential growth in human donor milk use. Guidelines for milk banking have been published in many countries including Australia, France, India, Italy, Spain, Switzerland, the United Kingdom and the nonprofit organization PATH. The European Milk Bank Association and the Human Milk Banking Association of North America have also published recommendations for milk banks throughout Europe and North America, respectively. Although there is variability among these guidelines, there is general consensus on quality control measures required to provide a supply of safe donor milk. These measures include effective donor screening, safe collection, transport and storage of milk, standardized pasteurization and bacteriological testing. Operational considerations are also critical, such as appropriate training for staff, equipment maintenance and cleaning, protocol and record keeping and inspection and accreditation. Clearly delineating these key quality control measures provides an excellent foundation for establishing international guidelines. Acceptable modifications must be established for low‐ and middle‐income countries that do not have sufficient resources; overly burdensome guidelines may make establishing a milk bank unnecessarily prohibitive. This review presents a summary of current best practices for human milk banking.

## Key messages

- Human donor milk is a vital source of nutrition for preterm very low birth weight infants when there is an insufficient supply of their own mother's/parent milk.
- Human milk banks follow best practices within their own jurisdictions, including for screening donors and for processing and distributing human donor milk.
- Internationally agreed upon guidelines for the operation of a milk bank that are modifiable to a country's resources would facilitate quality control.

## 1 INTRODUCTION

The aim of this report is to describe the technical aspects of human donor milk banking from donor screening to donor milk processing and distribution. Throughout the report, milk from a mother or parent for their own child is referred to as the gender inclusive mother's/parent milk (Dodgson et al., 2022). Human milk is referred to as the collective of mother's/parent milk and donor milk. What follows is a composite of published guidelines from Australia (Australian Government, 2022), Brazil (Angencia National de Vigilancia Sanitaria, 2008; Gutierrez & de Almeida 1998), France (FHMBA, 2007), India (Bharadva et al., 2014), Italy (Italian Association of Human Milk Banks et al., 2010), Spain (Calvo et al., 2018), Switzerland (Frischknecht et al., 2010), the United Kingdom (NICE, 2010), the nonprofit organization PATH (PATH, 2019), the European Milk Bank Association (Weaver et al., 2019) and the Human Milk Banking Association of North America (HMBANA, 2024). The reader is referred to the associated publications in this supplement for details of a framework to support global milk banking including policy development, sustainability, regulatory requirements and a comparison to established medical products of human origin programs (Israel‐Ballard et al., 2024; Weaver, 2024).

### 1.1 Donor milk

Donor milk is defined as human milk in excess of an infant's current and future needs that is donated by a parent for use by another infant (PATH, 2019). Donor milk is offered voluntarily, typically without payment to the donor, and is provided to the recipient infant prioritised by clinical necessity. Recipients are typically preterm and/or low birthweight infants. Donor milk is not an alternative to mother's/parent milk but is instead used as an alternative to formula serving as a bridge to ensure an exclusively human milk diet, as the family is provided lactation support to build milk supply (PATH, 2019).

### 1.2 Human milk bank

A human milk bank is a service established to recruit and screen human milk donors, collect and process donated milk and distribute the milk to meet infants' specific needs for optimal health. Ideally, human milk banks champion optimal lactation support within their community.

### 1.3 History of milk banking

The first documented human milk bank opened in 1909 in Austria (Barrett & Hiscox, 1939; Jones, 2003), followed by the United States (1910) (Talbot, 1928) and Germany (1919) and many others worldwide (Springer, 1997). Early banks were established in response to the diminishing number of wet nurses. A decline in the use of donor milk occurred in the 1950s and 1960s as artificial formula feeding became common. Once the shortcomings of artificial formula feeding were recognised, milk banking increased in popularity, peaking in the 1980s (Bednarek, 1982; Sauve et al., 1982). The HIV/AIDS crisis of the mid‐1980s resulted in the closure of many banks and a cessation in donor milk research. A resurgence in milk banking began in early 2000s with the ability to thoroughly screen donors and the safety of donor milk being further assured through standardized donor milk pasteurization techniques. In current neonatal care, there has been an unprecedented growth in donor milk banking (HMBANA, 2022). There remains a comparative discrepancy in the development of milk banks in low‐ and middle‐income countries (PATH, 2022).

## 2 MILK COLLECTION

With high interindividual and intraindividual variability of milk, the collection method has the potential to impact the nutrient content of the milk (Azad et al., 2021). Milk can be extracted from the breast using manual expression or a breast pump. Advantages of hand expression are that it is inexpensive, does not require equipment, can be easily taught and some find hand expression more comfortable than a pump (Becker et al., 2015). Both hand‐powered and electric pumps can be used to express milk. Manual pumps are less expensive, do not require power and have fewer parts than electric. Pumps can be a source of bacterial contamination if not cleaned properly between uses. The most suitable method for milk expression is dependent on individual circumstances and may be tailored to the purpose for not feeding directly at the breast (Becker et al., 2015; Meier et al., 2016).

Given that the concentration of nutrients changes from the beginning to the end of a feed, collecting all milk from a full breast will yield a different quantity of nutrients compared to a partial expression. For example, the lipid concentration increases as the breast is emptied. Drip milk is passively released during a feed from the nonnursing breast or collected in shells between feeds. Drip milk has been found to contain less fat and other nutrients than hand expressed milk (Stocks et al., 1983). For this reason and the risk of contamination, drip milk is not recommended for donation.

## 3 MILK STORAGE

### 3.1 Collection containers

Containers for storing human milk must be designated as food grade to avoid chemical contamination (Jones, 2019). Acceptable containers include milk storage bags, baby bottles and food storage containers with tight fitting lids. While some banks use glass bottles, others do not due to risk of breakage and additional weight during transportation. Stainless steel containers, as used in India, are durable and easy to clean. The nutrient composition of milk, particularly fat, is affected by the type of container used (Janjindamai et al., 2013). Ideally, milk is stored in glass or hard plastic containers. Polyethylene bags can be used although fat adheres more readily to soft plastic, and there is a potential loss of up to 60% of immunoglobulin secretory immunoglobulin A (sIgA) (Garza & Nichols, 1984). Milk bags are more prone to spillage or tearing particularly if overfilled. For in‐hospital collection of milk, storage in plastic specimen containers (orange lids) is not recommended as the orange dye in the lid may leach into milk and may be unsafe (Blouin et al., 2014). All plastic containers should be bisphenol‐A (BPA) free, a chemical used in the manufacturing of plastic that is associated with a negative impact on human health. Further research is required in recommending milk containers as concerns have recently been raised for BPA replacements in plastic (Rubin & Seebacher, 2022).

### 3.2 Milk handling

Human milk is not sterile, and therefore, the use of clean but not necessarily sterile containers and lids is adequate for home collection (Pittard et al., 1991). Lids must seal the container well; baby bottle nipples/teats are not recommended as microbes may enter through the hole. In the case of collecting milk in the hospital environment for provision to high‐risk infants, it is recommended that milk storage containers be sterile (Cossey et al., 2011).

### 3.3 Labelling

Containers should be labelled with the collection date, donor ID number and a second identifier such as the donor's date of birth or name depending on local privacy recommendations. In‐hospital management of milk administration ideally involves the use of a barcoding system to decrease error and improve the ease of traceability.

### 3.4 Temperature considerations

Advice provided for human milk storage intended for donation is more prudent than that for milk intended for home use (PATH, 2019). For milk collected for *one's own infant*, freshly expressed milk is safe at room temperature for 4–6 h and at 15°C for 24 h (Pittard et al., 1991). At 4°C, human milk can be safely stored for 4–8 days. Breakdown of fat occurs at storage temperatures above −70°C, and samples stored at −20°C exhibit a reduction in fat over time (Schlotterer & Perrin, 2018). Milk that is intended for *donation* should be chilled immediately and frozen within 24 h of expressing with a new container used every 24 h to reduce the introduction of contaminants. Freezing for up to 9 months does not appreciably affect total protein, fat, lactoferrin, sIgA or osmolality but reduces pH, folate, vitamin C, and total bacteria count (Ahrabi et al., 2016; Bank et al., 1985). Whilst recommendations differ, in North America, milk is acceptable for donation for up to 3 months from expression if stored in a freezer that is part of a fridge‐freezer and for up to 6 months from expression if stored in a free standing −20°C freezer (HMBANA, 2024).

### 3.5 Milk transportation

A cold chain is required to transport donated milk to the milk bank to ensure maximum retention of nutrients and mitigate the risk of bacterial growth (PATH, 2019). Transportation methods vary considerably from country to country and even milk bank to milk bank. In Canada, tightly packed frozen milk is transported by courier in insulated coolers with frozen gel packs for up to 24 h. In Brazil, milk is picked up at homes by volunteer fire fighters and paramedics and transported in vehicles with refrigerated storage while in Israel, milk is picked up by vehicles from the shared blood and milk bank services. Using ice cubes is not recommended as they may promote faster thawing of the milk. In some cases, small amounts of dry ice may be preferable, when shipping overnight, small milk volumes or in high outside temperatures. Upon arrival at the milk bank, donor milk must be visually checked to ensure the milk is still frozen.

## 4 QUALITY CONTROL

The most significant measure is heat treatment (pasteurization) of milk, but selection of healthy donors, establishing optimal procedures for collecting and storing milk and conducting bacteriological testing further contribute to quality control (Tyebally Fang et al., 2021). Staff training, equipment maintenance, standardized protocols and effective record keeping also play a role in maintaining donor milk safety. Although there is variability among guidelines for milk banking from different countries, there is consensus on the quality control measures listed below amongst recommendations from Australia (Australian Government, 2022), Brazil (Angencia National de Vigilancia Sanitaria, 2008; Gutierrez & de Almeida, 1998), France (FHMBA, 2007), India (Bharadva et al., 2014), Italy (Italian Association of Human Milk Banks et al., 2010), Spain (Calvo et al., 2018), Switzerland (Frischknecht et al, 2010), the United Kingdom (NICE, 2010), the nonprofit organization PATH (PATH, 2019), the European Milk Bank Association (Weaver et al., 2019) and the HMBANA (HMBANA, 2024).

### 4.1 Donor screening

Selecting healthy, reliable donors is important in ensuring the safety of donor milk. Interested women are screened by trained staff for lifestyle and clinical history (e.g., smoking, drug use, risk factors for sexually transmitted infections, transfusions, travel) and undergo blood testing (Australian Government, 2022; FHMBA, 2007; HMBANA, 2024; Italian Association of Human Milk Banks et al., 2010; NICE, 2010; Weaver et al., 2019).

### 4.2 Milk expression and storage

Milk bank personnel advise donors on hygiene measures including handwashing, proper daily pump part disinfection and container selection. Milk should be immediately refrigerated at 4°C or directly frozen. Containers should be filled with consideration for expansion during freezing and frozen at −20°C within 24 h of expression.

### 4.3 Pasteurization

For nonprofit milk banks, heat treatment at 62.5°C for 30 min (Holder method) is the standard pasteurization of donor milk, typically conducted in a hot water bath. Milk temperatures are monitored and recorded, and milk is then rapidly cooled to 4°C. Vat‐processed (63°C × 30 min) or commercially sterilised (retort, 121°C × 5 min with 15 PSI) human milk is also available in North America from for‐profit companies. Other, less harsh methods of milk treatment are under active investigation but have not yet been adopted and include high hydrostatic pressure and UV‐C irradiation (Lima et al., 2017; Pitino et al., 2019). The European Milk Bank Association (EMBA) recommends a common set of parameters to evaluate new pasteurization technologies including microbial challenges for bacteria and viruses (Moro et al., 2019).

### 4.4 Bacteriological testing

Recommendations for bacteriological screening of pooled milk before pasteurization vary including which bacterial thresholds warrant discarding milk. Postpasteurization testing is mandatory with an aliquot of milk from every batch cultured. The presence of any bacterial growth is not acceptable (equivalent to <10^3^ CFU/L according to most culture protocols). Any presence of *Bacillus cereus* or other heat‐forming spores either prepasteurization or postpasteurization is not allowable (Australian Government, 2022; FHMBA, 2007; HMBANA, 2024; Italian Association of Human Milk Banks et al., 2010; NICE, 2010; Weaver et al., 2019).

### 4.5 Facility operations

Milk banks require high‐quality, well‐maintained, clean equipment (freezers, pasteurizers, thermometers) that is regularly calibrated (every 6 months). Ongoing training of staff who oversee the handling of donor milk is critical to the maintenance of a safe donor milk supply.

Adherence to key protocols and quality control measures is guided by standards of practice set by local associations, such as HMBANA and EMBA. Many milk banks employ the HACCP system (hazard analysis and critical control points) to develop a rigorous safety analysis plan (Hartmann et al., 2007). The maintenance of quality control records such as pasteurization temperature logs and the ability to trace donor milk are essential safety elements. Also essential is a process for regular inspection and accreditation of milk banks through the appropriate oversight regulatory bodies and/or milk banking associations (Angencia National de Vigilancia Sanitaria, 2008; Australian Government, 2022; FHMBA, 2007; HMBANA, 2024; Italian Association of Human Milk Banks et al., 2010; NICE, 2010; Weaver et al., 2019).

## 5 EFFECTS OF PROCESSING ON BIOLOGICAL ACTIVITY OF HUMAN MILK

To ensure the safety of donor milk, nearly all milk banks worldwide have established pasteurization protocols. Exceptions are Norway, where donor milk is provided raw, and Germany, where some milk banks offer both raw and pasteurised milk (EMBA, 2023).

### 5.1 Holder pasteurization

Milk is thawed in a manner where it is maintained at or below 7.2°C and then pooled by combining the milk of a single donor or of three to four donors at various stages of lactation. Some milk banks take a sample from each pool for bacteriological testing; milk is then aliquoted into individual containers. These are heat processed using Holder pasteurization which consists of submerging containers in a shaking water bath (preheated to 62.5°C) or a specially designed pasteurizer for human milk. A control bottle fitted with a calibrated thermometer and containing the same quantity of human milk (potentially milk unsuitable for donation purposes to avoid wastage) follows the entire process. Once the temperature of the control bottle achieves 62.5°C, the heat treatment proceeds for 30 min. At the end of the pasteurization period, milk is immediately rapidly cooled to 4°C, either in the pasteurizer, in specialised cooling equipment, or in ice baths. An aliquot of processed milk from each batch is cultured for bacteria. The presence of any bacteria in the heat processed milk is unacceptable, and the milk is either discarded or used for research purposes (Australian Government, 2022; FHMBA, 2007; HMBANA, 2024; Italian Association of Human Milk Banks et al., 2010; NICE, 2010; Weaver et al., 2019). Holder pasteurization is ineffective against bacterial spores (e.g., *B.cereus*), of which some can be heat activated and may therefore appear in higher concentrations postpasteurization. Contamination of donor milk with spore‐forming bacteria may result in significant loss of pasteurized milk and varies according to geographical location and season (Adjide et al., 2022).

### 5.2 Effect of holder pasteurization on milk composition

Holder pasteurization per se does not substantially affect the macronutrient composition of donor milk, although transferring milk from container to container results in a measurable loss of the lipid fraction of the milk. The course of storing and processing donor milk typically involves five container changes and two freeze–thaw cycles. These manipulations and the pasteurization process can all impact milk composition. A summary of pasteurization effects on human milk is presented in Table 1. Despite heat‐induced alterations to the bioactive components of human milk, pasteurized milk maintains a degree of bacteriostatic and immune‐stimulating properties. The partially preserved biological activity likely contributes to the improved outcomes observed in preterm infants fed donor milk, compared to infant formula. Obliteration of all cellular activity (T cells, B cells, macrophages, neutrophils) occurs, along with a reduction in antibody immunoglobulin A (IgA reduced by 0%–48%), lactoferrin (reduced by 57%–80%) and lysozyme (reduced by 0%–60%) (Ewaschuk et al., 2011; Peila et al., 2016). Both bile salt‐stimulated and lipoprotein lipase are fully inactivated through Holder pasteurization, an important finding given the dependence of the preterm infant on these enzymes for fat absorption (Pitino et al., 2019). Tight control of mean temperature during pasteurization decreases the percentage of loss of lactoferrin, lysozyme, and IgA, compared to a broader temperature range (Buffin et al., 2018). Enhancements in milk pasteurizing equipment have improved the consistency and quality assurance of holder pasteurized human milk (Moro et al., 2019).

**Table 1:** Effect of Holder pasteurization on human milk component concentrations.

Component	Maintained (>50%)	Reduced (10%–50%)	Inactivated (<10%)
Macronutrients	Carbohydrate Protein and amino acids Total fat and fatty acids		
Micronutrients	Calcium Copper Iron Magnesium Phosphorus Potassium Sodium Zinc		
Vitamins	Vitamin A Folate Vitamin B2, B3, B5, B6, B7, B12 Vitamin C Vitamin D Vitamin E		
Immune components	IGF‐1, IGF‐2 IGF‐BP2,3 IFN‐ϒ IL‐1β, IL‐2, IL‐4, IL‐5, IL‐8, IL‐10, IL‐12p70, IL‐13, IL‐17 Gangliosides TGF‐α	CD14 (soluble) IgA, sIgA IgG IL‐2 Lactoferrin Lactoferrin‐iron binding capacity Lysozyme	IgM T cells B cells Macrophages Neutrophils
Metabolic components	Adiponectin Amylase Insulin Epidermal growth factor Heparin‐binding growth factor MCP‐1 TGF‐α, TGF‐β	Erythropoietin Hepatocyte growth factor	Bile salt‐dependent lipase Lipoprotein lipase Alkaline phosphatase

*Note*: Adapted from Ewaschuk 2011 (Ewaschuk et al., 2011) and Peila 2016 (Peila et al., 2016).

Abbreviations: CD‐14 ‐ cluster of differentiation 14; IFN, interferon; IgA, immunoglobulin A; IGF, insulin‐like growth factor; IgG, immunoglobulin G; IL, interleukin; MCP‐1, monocyte chemoattractant protein 1; sIgA, secretory immunoglobulin A; TGF, transforming growth factor.

### 5.3 Other pasteurization methods

Shelf‐stable donor milk (retort processing [121°C, 15 PSI, 5 min]) is commercially available in the United States. One study of shelf stable donor milk showed that it had lower total protein and fat content, compared to Holder pasteurized samples while another group observed increased protein content in shelf stable milk (Lima et al., 2017; Meredith‐Dennis et al., 2018). Many immune modulating molecules (IgA, immunoglobulin G [IgG], IgM, lactoferrin, lysozyme, α‐lactalbumin, α‐antitrypsin, and oligosaccharides) are also lower in shelf stable human milk compared to holder pasteurized human milk. An advantage of this heat treatment is its effectiveness against *B.cereus*, an environmental pathogen, whose spores are resistant to Holder pasteurization.

High‐temperature short‐time (HTST) thermal processing has been used in the dairy milk industry since the 1930s and involves forcing milk between plates or pipes that are heated to 72°C, for 5–15 s. This method holds some promise in the treatment of donor milk and appears to be as effective as Holder pasteurization in ensuring microbiological safety. Several milk components are better preserved by HTST treatment compared to Holder, including lactoferrin, B vitamins, lipase, sIgA and some cytokines; however, levels are often well below those of untreated milk (Peila et al., 2017, Pitino et al., 2019). A recent report studied not only the reduction in levels of bioactive molecules following HTST processing but also the functionality of key molecules. They found a 52% and 81% reduction in activity of lactoferrin and bile salt stimulated lipase, respectively, when comparing HTST processed milk to untreated donor milk (Kontopodi et al., 2021). Like holder pasteurization, HTST is not effective against bacterial spores, and there is some evidence it may not be effective against nonenveloped viruses such as hepatitis A (Peila et al., 2017). There are currently no devices available commercially for the HTST processing of human milk.

High hydrostatic pressure (HHP) is widely used in the food industry for juices, beverages, meats and other food products. It employs high hydrostatic pressure (300–800 MPa), with or without heat, for short periods of time (5–10 min) to inactivate pathogens. At pressures below 600 MPa, especially in the absence of heat, HHP is thought to better preserve the bioactive components in human milk including lactoferrin, lysozyme and bile salt simulated lipase (Peila et al., 2017; Pitino et al., 2019) and to inactivate spores when used in combination with low heat (Demazeau et al., 2018). The effectiveness of HHP on viruses that may be present in human milk has begun to be explored (Pitino et al., 2022). HHP equipment used commercially may be prohibitively large and expensive for most milk banks; however, smaller pilot plant versions for research purposes are available.

Ultraviolet‐C pasteurization uses short‐wavelength ultraviolet radiation to disrupt the nucleic acids in microorganisms. It is difficult to treat human milk with UV‐C due to its opacity and therefore limited capacity for photon penetration. Experimentally, the treatment appears to better preserve the bioactivity of some milk components compared with Holder pasteurization and significantly reduces total bacterial load, but its effectiveness in destroying viruses is as yet unknown (Peila et al., 2017; Pitino et al., 2019). No commercially available equipment currently exists to permit the use of this method in a human milk bank.

## 6 SCREENING AND SELECTION OF HUMAN MILK DONORS

Many jurisdictions consider donor milk to be a food and regulate it accordingly, while other jurisdictions regulate donor milk as a biological tissue. Regardless of the framework, milk banks may consider using screening methodologies for potential donors equivalent to those used for blood donation. Although there are slight differences in screening protocols in published milk banking guidelines, there is generally a core set of eligibility criteria that a person must meet to donate milk (Australian Government, 2022; FHMBA, 2007; Frischknecht et al., 2010; HMBANA, 2024; Italian Association of Human Milk Banks et al., 2010; F. Jones, 2019; PATH, 2019; Weaver et al., 2019). Of note, mothers/parents who do not meet screening criteria are typically able to continue breastfeeding their own infant and should be encouraged to do so.

### 6.1 Training interviewers

Effective screening relies on the skill of the interviewers who need to be familiar with the guidelines governing their milk bank (e.g., HMBANA Standards for Donor Human Milk Banking [HMBANA, 2024] or EMBA Recommendations for the Establishment and Operation of Human Milk Banks in Europe) (Weaver et al., 2019). They should be familiar with the rationale for each inclusion and exclusion criteria. Screening staff should know to whom they may address questions regarding clarification of inclusion and exclusion criteria (e.g., milk bank medical director). Training of interviewers should include an assessment for skill level and should be repeated regularly with enhanced training and scope for dialogue around difficult scenarios (e.g., bereavement, medications contraindicated during donation, off taste milk, infant illness, infant feeding intolerance, mixed feeding with artificial formula milk).

### 6.2 Informed consent

Informed consent describing the intended use of the milk, including research, must be obtained from all donors, with documents securely stored (Australian Government, 2022; HMBANA, 2024; Weaver et al., 2019).

### 6.3 Screening interview

The screening interview can be oral and/or written and aims to assess the following:

1. **Donor health and donor's infant health.** Statements of health and known medical risks signed by the donor's healthcare provider +/‐ the infant's healthcare provider give additional assurance.
2. **Medications.** Includes prescription drugs, over‐the‐counter medications, vaccines and herbal remedies.
3. **Lifestyle**. The donor and her partner's lifestyle including smoking, alcohol and recreational drug use, tattoos and piercings, environmental exposures and risk factors for infectious disease (e.g., travel to endemic areas for viral diseases).
4. **Milk collection.** The types of collection containers and freezers used to store milk as well as whether milk was expressed for donation before contacting the milk bank (this milk may be used in separate batches).

Documented communication about the donor's health continues regularly throughout the donation period (e.g., every 2 months) and is further captured at the time of each donation.

### 6.4 Serology

All donors must have their blood tested in a certified laboratory within 6 months of the first donation and this may be repeated every 6 months. Best practice demands serology testing at the time of recruitment. Required blood tests include:

1. HIV‐1, HIV‐2
2. HTLV‐1, HTLV‐2
3. Hepatitis B
4. Hepatitis C
5. Syphilis

Some guidelines include nucleic acid testing for hepatitis B and C and HIV.

### 6.5 Exclusion criteria

Some lifestyle or health factors warrant that a woman be excluded from donating her milk.

Exclusion criteria include:

1. Smoking (including second‐hand smoke), other nicotine use (vaping, nicotine replacement therapy).
2. Alcohol intake beyond recommended levels (no consensus on safe amounts, North American guideline is no more than 1.5 oz hard liquor, 12 oz beer or 5 oz wine in 24 h with 12 h of exclusion after alcohol ingestion; PATH and UK guidelines are 1–2 units of alcohol once or twice a week).
3. Recreational drug use in the last 12 months (marijuana is currently contentious as it is legal in some countries, but the time course until it is cleared from milk post usage remains uncertain).
4. Blood transfusion, accidental needle stick, piercings with nonsingle‐use instruments and tattoo or permanent makeup at a nonregulated site within the past 3–6 months.
5. Organ transplant within the last 12 months (ongoing immunosuppressant therapy is an additional contraindication).
6. Medications beyond allowable list (HMBANA provides such a list for example).
7. Chronic infections (HIV, HTLV, TB), history of hepatitis B or C, history of leukemia or lymphoma are lifelong contraindications.
8. Treatment for cancer within the last 3 years.
9. Sexual partner within 12 months who is at risk for HIV, HTLV or hepatitis.
10. Risk of Creutzfeldt‐Jakob disease.
11. Exposure to Ebola within 28 days.
12. The following vaccines within the last 8 weeks: yellow fever, oral polio, oral typhoid, smallpox.

### 6.6 Temporary exclusion

Temporary exclusion from donating milk may periodically be required. Possible reasons for temporary disqualification include breast or other infections, consumption of drugs, alcohol or nonallowable medications. In the absence of available evidence for new scenarios, milk banks must have local expertise that can be consulted (e.g., serology and microbiology oversight, pharmacological expertise, and neonatal input).

## 7 PANDEMICS AND MILK BANKING

At the outset of the COVID‐19 pandemic, concerns arose about protecting milk banking in view of the previous history of most human milk banks closing in the 1980s for fears of HIV transmissibility (Shenkar et al., 2021). Scientific evidence quickly showed that transmission of the SARS‐CoV‐2 virus into human milk is rare and may come from contamination of the milk postexpression (Krogstad et al., 2021). It was also shown that antibodies to SARS‐CoV‐2, both IgA and IgG, are present in human milk following either vaccination or infection, although the time course of antibody appearance and disappearance has not been fully elucidated (Perez et al., 2022). Further, human milk is neutralizing to SARS‐CoV‐2 with the mechanism not solely antibody mediated. Finally, Holder pasteurization has been shown to fully neutralize this virus (Unger et al., 2020). A recent survey of North American milk banks documented staffing and organizational challenges throughout the pandemic, but milk donations tended to increase (Cohen & Cassidy, 2021). This may be secondary to donors being at home and not requiring their surplus milk for their own children or else having more time to express milk if working from home.

## 8 HUMAN MILK DONORS

A recent systematic scoping review of what is known about milk donors documented limitations in present knowledge of donors along with heterogeneity in the variables reported in the 28 studies identified (Gutierrez & de Almeida, 1998). Notable gaps in the literature identified by this review include barriers to milk donation and motivation to donate milk including motivation to informally share or to sell milk.

### 8.1 Characteristics of milk donors

A study from 2003 reported data from 103 milk donors from France. Donors were women of average childbearing age, with strong home support. These findings are consistent with those from the United States where women with the highest breastfeeding rates and likelihood to be milk donors tend to be married, college educated and of high socioeconomic status (CDC, 2022). The association between maternal schooling and donation was not observed in a cross‐sectional study from Brazil (Meneses et al., 2017).

### 8.2 Motivations of donors

Reported primary motivators for women to donate milk include an altruistic feeling and having an excess of milk (Gutierrez dos Santos & Perrin 2021; Martinez‐Sabater et al., 2014). Receiving support with milk expression, encouragement to donate milk from knowledgeable health professionals and the availability of infrastructure to facilitate convenient donation were also supporting factors. Consistent with other reports, a qualitative study by Perrin et al. concluded that the reasons women choose to donate to peers rather than to milk banks is based, in part, on an unexpected surplus of milk, the absence of healthcare providers as a source of information about milk banking and misconceptions about milk bank costs and convenience (Perrin et al., 2016). Further, peer‐to‐peer sharers value the personal experience of assisting another family. The authors suggested that donor education campaigns may improve the likelihood of women choosing to donate excess milk to a milk bank rather than peer‐to‐peer sharing.

### 8.3 Experiences of milk donors

One qualitative study of 12 mothers identified four themes that represented their experience as milk donors (Candelaria et al., 2018). They felt that their contribution was helping others, instilled feelings of pride, felt gratitude towards nursing staff who had encouraged them and had a desire to talk about their experiences of donating milk.

Milk donation following bereavement is emerging as an important donation that may benefit both the milk bank and the bereaved parent(s) as it may be conceptualised as part of the grief ritual in some ways comparable to organ donation (Oreg, 2020). Mothers with a pregnancy loss as early as 17 weeks gestation may go on to lactate for the purpose of donation. A case report of two American women who opted to donate their milk with the knowledge of a life‐threatening fetal diagnosis reported a desire to help others and connection with their infant after they passed as key motivating factors (Cole et al., 2018). To provide patient‐centred lactation care in bereavement requires a planned care pathway that includes healthcare providers who are knowledgeable in the full spectrum of options available to bereaved parents (Noble‐Carr et al., 2021).

## 9 ETHICAL ISSUES IN HUMAN MILK BANKING

### 9.1 Medical decision making

The use of donor milk in clinical settings depends on milk availability, clinician knowledge and experience with donor milk and individual patient needs. Clinicians have an obligation to be aware of the best scientific evidence and to communicate their knowledge in an unbiased, noncoercive manner. Clinicians must remain vigilant of conflicts of interest that may affect their clinical decision‐making in the event of research‐related relationships with for‐profit companies that sell formula, donor milk or nutrient fortifiers.

#### 9.1.1 Informed consent and confidentiality

Both donors and recipients must provide informed consent. The informed consent requires that the parents of infants be provided with current information on feeding options. It is important to underscore that while donor milk is superior to formula for VLBW infants, a mother's own milk provides by far the best nutrition.

### 9.2 Payment for milk

Globally, most not‐for‐profit milk banks do not financially compensate their donors (Australian Government, 2022). Most milk banks charge a processing fee to those ordering donor milk to cover processing costs. In Austria and Norway, donors are reimbursed their expenses. In Canada, it is illegal to sell any body part or fluid, and so, donors are never financially compensated. Paying donors for their milk creates the potential for donors to adulterate milk, to compromise their own babies' health by donating too much milk and may attract unhealthy donors. The issue of payment has further ethical complexity when the milk bank receiving the milk is operating for profit.

### 9.3 Allocating a limited supply of donor milk

There is limited availability of pasteurized human donor milk. Using a probabilistic economic model to optimize the allocation of donor milk, a South African group evaluated scenarios in which infants of varying birthweight were exclusively fed formula or donor milk for 14 or 28 days (Taylor et al., 2018). Prioritising infants in the lowest birthweight groups resulted in the most lives saved. These findings are consistent with HMBANA's suggested priorities for dispensing donor milk, with preterm sick infants having the highest priority followed by well preterm infants and then infants less than 12 months of age with medical conditions likely to respond to donor milk therapy (HMBANA, 2024).

### 9.4 Religious considerations

Several aspects of Jewish law have implications for observant Jews in the provision of donor milk and in expressing milk for VLBW infants (Kassierer et al., 2014). Breastfeeding is valued by Jewish tradition; the Talmud recommends 24 months of breastfeeding. Human milk is exempt from Jewish dietary laws and is permitted from any woman, whether keeping kosher or not. An observant Jewish family, however, may have metaphysical concerns about providing donor milk from a nonkosher keeping mother to their child. The preservation of life (*pikuach nefesh*) is a paramount law in Judaism that supersedes all dietary law and provides a powerful argument for the provision of donor milk to the VLBW infant (Kassierer et al., 2014).

The use of donor milk may also raise religious concerns for families of Muslim faith (El‐Khuffash & Unger, 2012; Subudhi & Sriraman, 2021). The primary concept surrounding the use of donor milk is that of ‘milk kinship’. This law establishes kinship between infants who have been nursed by the same mother. For some Muslim families, this may be considered for the recipients of donor milk signifying that the children of the donor mother are regarded as siblings of the recipient infant and are not permitted to marry. The European Council for Fatwa and Research has issued a decree that providing donor milk to infants in the NICU does not meet the law of milk kinship, as it does not involve the act of suckling and does not involve a fulfilling feed as there are typically several donors' milk in one batch of donor milk. The preservation of health is also a powerful argument in favor of donor milk for preterm infants of Muslim families. Healthcare providers must have a good understanding of Islamic milk kinship laws to provide the best counselling for their families of Muslim faith (El‐Khuffash & Unger, 2012). Human milk banks have now been established in a number of predominantly Islamic countries (e.g., Iran, Malaysia).

## 10 CONCLUSION

Current interest in milk banking has exponentially grown, including both the volume of donor milk being dispensed and research in the field. The need for international guidance on milk banking procedures and future research is of timely importance.

## AUTHOR CONTRIBUTIONS

Sharon L. Unger and Deborah L.O'Connor contributed equally to the writing and editing of this paper. They are equally responsible for content.

## CONFLICTS OF INTEREST STATEMENT

Sharon Unger is the medical director for the Rogers Hixon Ontario Human Milk Bank and a co‐primary investigator for the MaxiMoM: Maximizing Mother's Milk for Preterm Infants program of research. Deborah O'Connor is the past chair of the Advisory Board for the Rogers Hixon Ontario Human Milk Bank and a co‐primary investigator for the MaxiMoM: Maximizing Mother's Milk for Preterm Infants program of research.

## Data Availability

Data sharing is not applicable to this article as no new data were created or analysed in this study.
